# Co-infection of sexually transmitted pathogens and Human Papillomavirus in cervical samples of women of Brazil

**DOI:** 10.1186/s12879-017-2835-5

**Published:** 2017-12-15

**Authors:** Aline Teixeira Amorim, Lucas Miranda Marques, Guilherme Barreto Campos, Tássia Neves Lobão, Vanesca de Souza Lino, Ricardo Cesar Cintra, Maria Antonieta Andreoli, Luisa Lina Villa, Enrique Boccardo, Antonio Carlos Ricardo Braga Junior, Rossana Verónica Mendoza López, Djanilson Barbosa dos Santos, Gerson Maciel de Souza, Carla Cristina Romano, Jorge Timenetsky

**Affiliations:** 1 Departamento de Microbiologia, Instituto de Ciências Biomédicas, Universidade de São Paulo, ICB/USP, Avenue Prof. Lineu Prestes n°1374 - Butantã, São Paulo, SP 05508-900 Brazil; 2 Instituto Multidisciplinar em Saúde/Campus Anísio Teixeira, Universidade Federal da Bahia – IMS/CAT-UFBA, Vitória da Conquista, Brazil; 3 Departamento de Bioquímica, Universidade de São Paulo, USP, São Paulo, Brazil; 4 Faculdade de Ciências Médicas da Santa Casa de São Paulo, FCMSCSP, A.C. Camargo Cancer Center, São Paulo, Brazil; 5 Faculdade de Medicina da Universidade de São Paulo, Instituto do Câncer do Estado de São Paulo, São Paulo, Brazil; 6 Instituto do Câncer do Estado de São Paulo, São Paulo, Brazil; 7 Universidade Federal do Recôncavo da Bahia, Santo Antônio de Jesus, Brazil; 8 Centro de Oncologia Dr. Geraldo Mattos de Sá, Vitória da Conquista, Brazil; 9 Universidade Estadual de Santa Cruz (UESC), Campus Soane Nazaré de Andrade, Ilhéus, Brazil

**Keywords:** Cervical cancer, HPV, *U. Parvum*, Sexually transmitted infections

## Abstract

**Background:**

Some sexually transmitted infectious agents, such as *Chlamydia trachomatis* and Herpes simplex, cause local inflammation, and could contribute to Human Papillomavirus (HPV) and cervical lesion progression. Thus, the aim of this study was to determine any association between the presence of microorganisms of gynecological importance, sexual behavior, clinical and demographical variables to the development and progress of cervical lesions.

**Methods:**

One hundred and thirty-two women between 14 and 78 years and living at Vitória da Conquista, Bahia, Brazil, were included (62 individuals with cervical lesions and 70 without lesions). They answered a questionnaire to provide data for a socioeconomic and sexual activity profile. Samples of cervical swabs were collected and analyzed by PCR to detect genital microorganisms and HPV. Quantitative PCR was used to detect and quantify *Ureaplasma urealyticum* and *Ureaplasma parvum*. Univariate and multiple logistic regression were performed to measure the association with the cervical lesions, and an odds ratio (OR) with 95% confidence intervals (95%CI) were calculated. The Mann-Whitney U test was also used to compare the microorganism load in the case and control groups. The significance level was 5% in all hypotheses tested.

**Results:**

Cervical lesions were associated with: women in a stable sexual relationship (OR = 14.21, 95%CI = 3.67–55.018), positive PCR for HPV (OR = 16.81, 95%CI = 4.19–67.42), *Trichomonas vaginalis* (OR = 8.566, 95%CI = 2.04–35.94) and *Gardnerella vaginalis* (OR = 6.13, 95%CI = 1.53–24.61), adjusted by age and qPCR for *U. parvum*. *U. parvum* load showed a statistical difference between the case and control groups (*p*-value = 0.002).

**Conclusion:**

Variables such as stable relationship, HPV, *T. vaginalis, G. vaginalis* were associated with cervical lesions in epidemiological studies. *U. parvum* load was higher in woman with cervical lesions compared with women without lesions. Additional studies are needed to better understand the role of these factors in cervical lesion development.

**Electronic supplementary material:**

The online version of this article (10.1186/s12879-017-2835-5) contains supplementary material, which is available to authorized users.

## Background

Cervical cancer is the fourth most common cancer among women in Brazil and seventh worldwide [1]. The highest disease incidence occurs in the least developed regions. Worldwide, in 2012, 528,000 new cases were detected and approximately 266,000 deaths occurred, accounting for 7.5% of all deaths of women by cancer [1]. In Brazil, 15,590 new cases of cervical cancer were reported in 2014, with an incidence of approximately 15 cases per 100,000 women [2]. In the Northeast of Brazil, cervical cancer ranks second (18.79/100,000) among all other cancers in Brazil, and in Bahia State 1300 new cases were estimated in 2014, with an average rate of 16.78/100,000 [2].

The development of cervical lesions depends on infection by high risk Human Papillomavirus (HPV), such as HPV-16 and HPV-18, and other factors. These HPV types express the oncoproteins E6 and E7, which interfere with cellular machinery causing cell immortalization and transformation. HPV-related carcinogenesis depends on different factors such as HPV type, virus persistence, sustained viral oncogene expression, viral load and viral genome integration [3].

As in other cancers, cervical cancer is also multifactorial. In addition to identifying HPV as the main etiological agent, additional risk factors such as use of oral contraceptives, smoking, early onset of sexual activity, multiple partners and chronic inflammation due to co-infection with other microorganisms have been strongly associated with cervical lesion progression [3–5].

Cervical inflammation may be associated with high-grade lesions and may be a cofactor for high-grade cervical lesions in women infected with oncogenic HPV. In other cancer types, independent of the causal agent, chronic inflammation exposes the tissue to constant genotoxic damages. In cervical cancer, chronic inflammation added to the initial changes caused by high-risk HPV, may contribute to viral persistence and disease progression [6, 7].

Some sexually transmitted infectious agents, such as *Chlamydia trachomatis* and Herpes simplex, cause local inflammation, and could contribute to HPV and cervical lesion progression [5]. Similarly, other microorganisms of gynecological importance such *Gardnerella vaginalis* and *Trichomonas vaginalis*, as well the species of *Mollicutes* (wall-less bacteria) are included in this context. These wall-less bacteria have been described for over 30 years as microorganisms related to urogenital disorders. Some species are the main cause of non-gonococcal and non-chlamydial urethritis (NGU-NC), as in the case of bacterial vaginosis (BV). *Mollicutes* may induce the release of cytokines such as IL-1, IL-6 and TNF-α and chemokines and may cause intense inflammation. This response has been suggested as a possible risk factor for developing precancerous lesions [8–10]. The combination of factors that play a role in a disease almost always requires studies to determine the precise contribution of each factor in a complex dynamic. Cervical cancer as such, is also a disease that, although it requires infection with certain HPV types to develop, involves multiple factors that are not fully understood. Studies establishing an association between *Mollicutes* and HPV may help better understand the role of these bacteria in developing cervical cancer. Therefore, the aim of this study was to analyze the existence of an association between the presence of microorganisms of gynecological importance, sexual behavior, clinical and demographic variables, and the development of cervical lesions in women from Vitória da Conquista city, Bahia, Brazil.

## Results

### Detection of sexually transmitted microorganisms for each cytological profile

From 62 women in the case group, 18 (29.0%) had low-grade squamous intraepithelial lesion (LSIL) described also as Cervical Intraepithelial Neoplasia (CIN I), 16 (25.8%) had high-grade squamous intraepithelial lesion (HSIL)/(CIN II) and 28 (45.2%) had HSIL/CIN III/in situ carcinoma. The detection of the afore mentioned microorganisms by PCR and qPCR methodologies was determined in samples of the case and control groups. The prevalence of the targeted sexually transmitted microorganism was calculated (Table 1).

**Table 1:** Detection of microorganisms included in the study of cervical samples

	Description of results from gynecological examinations					
Microorganisms	With cervical lesions (Cases)				Without cervical lesions (Controls)	Total^a^ *n* = 132
	Low grade (CIN I)	Intermediate (CIN II)	High grade (CIN III or in situ carcinoma)	Total		
	*n* = 18	*n* = 16	*n* = 28	*n* = 62	*n* = 70	
	n (%)	n (%)	n (%)	n (%)	n (%)	n (%)
PCR						
HPV						
Positive	13 (72.2)	14 (87.5)	26 (92.9)	53 (85.5)	18 (25.7)	**71 (53.8)**
Negative	5 (27.8)	2 (12.5)	2 (7.1)	9 (14.5)	52 (74.3)	61 (46.2)
*Mollicutes*						
Positive	18 (100.0)	14 (87.5)	25 (89.3)	57 (91.9)	48 (68.6)	**105 (79.5)**
Negative	0 (0.0)	2 (12.5)	3 (10.7)	5 (8.1)	22 (31.4)	27 (20.5)
*U. urealyticum*						
Positive	1 (5.6)	3 (18.8)	1 (3.6)	5 (8.1)	3 (4.3)	**8 (6.1)**
Negative	17 (94.4)	13 (81.2)	27 (96.4)	57 (91.9)	67 (95.7)	124 (93.9)
*U. parvum*						
Positive	6 (33.3)	12 (75.0)	14 (50.0)	32 (51.6)	35 (50.0)	**67 (50.8)**
Negative	12 (66.7)	4 (25.0)	14 (50.0)	30 (48.4)	35 (50.0)	65 (49.2)
*U. parvum –* serotype 1						
Positive	0 (0.0)	1 (6.2)	0 (0.0)	1 (1.6)	9 (12.9)	**10 (7.6)**
Negative	18 (100.0)	15 (93.8)	28 (100.0)	61 (98.4)	61 (87.1)	122 (92.4)
*U. parvum* serotypes 3/14						
Positive	5 (27.8)	2 (12.5)	4 (14.3)	11 (17,.)	10 (14.3)	**21 (15.9)**
Negative	13 (72.2)	14 (87.5)	24 (85.7)	51 (82.3)	60 (85.7)	111 (84.1)
*U. parvum –* serotype 6						
Positive	0 (0.0)	1 (6.2)	0 (0.0)	1 (1.6)	17 (24.3)	**18 (13.6)**
Negative	18 (100.0)	15 (93.8)	28 (100.0)	61 (98.4)	53 (75.7)	114 (86.4)
*Chlamydia trachomatis*						
Positive	1 (5.6)	0 (0.0)	1 (3.6)	2 (3.2)	1 (1.4)	**3 (2.3)**
Negative	17 (94.4)	16 (100.0)	27 (94.4)	60 (96.8)	69 (98.6)	129 (97.7)
*Neisseria gonorrhoeae*						
Positive	0 (0.0)	0 (0.0)	0 (0.0)	0 (0.0)	15 (21.4)	**15 (11.4)**
Negative	18 (100.0)	16 (100.0)	28 (100.0)	62 (100.0)	55 (78.6)	117 (88.6)
*Trichomonas vaginalis*						
Positive	10 (55.6)	10 (62.5)	18 (64.3)	38 (61.3)	4 (5.7)	**42 (31.8)**
Negative	8 (44.4)	6 (37.5)	10 (35.7)	24 (38.7)	66 (94.3)	90 (68.2)
*Gardnerella vaginalis*						
Positive	15 (83.3)	15 (93.8)	27 (96.4)	57 (91.9)	37 (52.9)	**94 (71.2)**
Negative	4 (16.7)	1 (6.2)	1 (3.6)	5 (8.1)	33 (47.1)	38 (28.8)
qPCR						
*U. urealyticum*						
Positive	2 (11.1)	5 (31.2)	1 (3.6)	8 (12.9)	9 (12.9)	**17 (12.9)**
Negative	16 (88.9)	11 (68.8)	27 (96.4)	54 (87.1)	61 (87.1)	115 (87.1)
*2 U. parvum*						
Positive	12 (66.7)	15 (93.8)	22 (78.6)	49 (79.0)	41 (58.6)	**90 (68.2)**
Negative	6 (33.3)	1 (6.2)	6 (21.4)	13 (21.0)	29 (41.4)	42 (31.8)

^a^Prevalence in all populations studied in boldface

### Sexual and sociodemographic profile and sexually transmitted agents as risk factors for cervical lesions

After questionnaire analysis, we obtained the sociodemographic and sexual profiles of the women interviewed. The mean age of women from both case and control groups was similar, being 38.7 (SD = 11.6) and 37.6 (SD = 14.4) years, respectively. However, although the general mean age between case and control groups was similar, there was punctual differences in age distributions between the groups. We can observe that among the women without cervical lesion, 40% were less than 31 years old. In women with cervical lesions, a higher frequency was found among those aged 32–42 years (40.3%). This corroborates the fact that the development of cervical lesions are time-dependent and highlights the importance of this variable for the subsequent analyses. Table 2 shows the sociodemographic and sexual profile distribution of the women in the case and control groups.

**Table 2:** Factors related to presence of cervical lesions

Variables	Without cervix lesions	With cervix lesions	OR (Crude)			OR (Adjusted)^b^		
	*n* = 70 (%)	*n* = 62 (%)	P (Wald’s test)	OR	IC 95%	P (Wald’s test)	OR	IC 95%
	n (%)	n (%)						
Demographic and sexual profile								
Age								
≤ 31 years	28 (40.0)	16 (25.8)	0.161	1			1	
32–42 years	19 (27.1)	25 (40.3)	0.057	2.30	0.98–5.42	0.316	2.27	0.46–11.18
43 years or more	23 (32.9)	21 (33.9)	0.281	1.60	0.68–3.75	0.470	0.57	0.12–2.64
Residence								
Urban area	60 (85.7)	41 (66.1)		1				
Countryside	10 (14.3)	21 (33.9)	0.010	3.07	1.31–7.20			
Education level								
At least primary school completed	39 (55.7)	18 (29.0)		1				
Less than primary school	31 (44.3)	44 (71.0)	0.002	3.08	1.49–6.34			
In a stable relationship								
No	57 (81.4)	16 (25.8)		1			1	
Yes	13 (18.6)	46 (74.2)	<0.0001	12.01	5.51–28.87	< 0.001	14.21	3.67–55.02
N° of sexual partners in all life								
1	30 (42.9)	16 (25.8)	0.121	1				
2–5	34 (48.6)	38 (61.3)	0.057	2.10	0.98–4.50			
> 5	6 (8.6)	8 (12.9)	0.141	2.50	0.738–8.47			
Ethnic group declared								
White	17 (24.3)	13 (21.0)		1				
No white	53 (75.7)	49 (79.0)	0.650	1.21	0.53–2.75			
Condom use								
Always	12 (17.6)^a^	11 (17.7)	0.988	1				
Sometimes	15 (22.1)^a^	13 (21.0)	0.921	0.92	0.31–2.85			
Never/rarely	41 (60.3)^a^	38 (61.3)	0.981	1.01	0.40–2.56			
Contraceptive pills/hormone injection use								
No	44 (62.9)	43 (69.4)		1				
Yes	26 (37.1)	19 (30.6)	0.432	0.75	0.36–1.55			
Pregnancy								
0	6 (8.6)	3 (4.8)	0.425	1				
1–2	34 (48.6)	26 (41.9)	0.573	1.53	0.35–6.70			
≥ 3	30 (42.9)	33 (53.2)	0.294	2.20	0.51–9.58			
Abortion								
No	42 (60.0)	43 (69.4)		1				
Yes	28 (40.0)	19 (30.6)	0.264	0.66	0.32–1.36			
STA detection								
HPV								
Negative	52 (74.3)	9 (14.5)		1		< 0.001	1	
Positive	18 (25.7)	53 (85.5)	<0.001	17.01	7.01–41.23		16.81	4.19–67.42
*Mollicutes*								
Negative	22 (31.4)	5 (8.1)		1				
Positive	48 (68.6)	57 (91.9)	0.002	5.23	1.84–14.85			
*U. parvum* (qPCR)								
Negative	29 (41.4)	13 (21.0)		1			1	0.36–5.98
Positive	41 (58.6)	49 (79.0)	0.013	2.66	1.23–5.79	0.593	1.47	
*U. parvum* (PCR)								
Negative	35 (50.0)	30 (48.4)		1				
Positive	35 (50.0)	32 (51.6)	0.853	1.07	0.54–2.11			
*U. parvum* serotype 1								
Negative	61 (87.1)	61 (98.4)		1				
Positive	9 (12.9)	1 (1.6)	0.040	0.11	0.01–0.90			
*U. parvum* serotypes 3/14								
Negative	60 (85.7)	51 (82.3)		1				
Positive	10 (14.3)	11 (17.7)	0.589	1.29	0.51–3.29			
*U. parvum* serotype 6								
Negative	53 (75.7)	61 (98.4)		1				
Positive	17 (24.3)	1 (1.6)	0.004	0.05	0.007–0.40			
*U. urealyticum* (qPCR)								
Negative	61 (87.1)	54 (87.1)		1				
Positive	9 (12.9)	8 (12.9)	0.994	1.00	0.36–2.79			
*U. urealyticum* (PCR)								
Negative	67 (95.7)	57 (91.9)		1				
Positive	3 (4.3)	5 (8.1)	0.371	1.96	0.45–8.56			
*T. vaginalis*								
Negative	66 (94.3)	24 (38.7)		1		0.003	1	2.04–35.94
Positive	4 (5.7)	38 (61.3)	<0.001	26.13	8.43–80.98		8.566	
*G. vaginalis*								
Negative	33 (47.1)	5 (8.1)		1		0.011	1	1.53–24.61
Positive	37 (52.9)	57 (91.9)	<0.001	10.17	3.64–28.41		6.13	
*C. trachomatis*								
Negative	69 (98.6)	60 (96.8)		1				
Positive	1 (1.4)	2 (3.2)	0.501	2.30	0.20–26.00			
*N. gonorrhoeae*								
Negative	55 (78.6)	62 (100.0)		1				
Positive	15 (21.4)	0 (0)	0.998	0				

^a^two women did not declare using condoms

^b^adjusted for age and positivity in qPCR for *U. parvum.* The goodness of the adjustment was evaluated by Hosmer-Lemershow test (*p* = 0.568)

In the univariate logistic regression, some variables showed risk for developing cervical lesions. The following sociodemographic and sexual variables showed as risk factors for cervical lesions: living in the countryside (OR = 3.07, 95% CI = 1.31–7.2), education level lower than elementary education (OR = 3.08, 95% CI = 1.49–6.34) and stable sexual relationship (OR = 12.61, 95% CI = 5.5–28.9). In regards to the microorganisms, positive PCR results to HPV (OR = 17.01, 95% CI = 7.01–41.3), *Mollicutes* (OR = 5.23, 95% CI = 1.84–14.84), *U. parvum* (qPCR) (OR = 2.67, 95% CI = 1.23–5.78), *T. vaginalis* (OR = 26.12, 95% CI = 8.43–80.96) and *G. vaginalis* (OR = 10.17, 95% CI = 3.64–28.41) presented as risk factors for cervical lesions. However, infection by *U. parvum* serotypes 1 (OR = 0.11, CI 95% = 0.01–0.9) and 6 (OR = 0.005, 95% CI = 0.01–0.4) appeared as protective factors (Table 2). In addition, we observed a higher detection of both *U. parvum* and *U. urealyticum* by qPCR compared with PCR.

All the variables that showed association in the univariate analysis were tested together to see if they contributed, together, to the risk of cervical injury. All variables that lost significance in the Wald’s test (*p* > 0.005) were withdrawn from the model one by one, except for the confounding variables or those of biological importance for the model (for example the variables age and presence of *U. parvum*). The most appropriate model was selected according to the goodness of fit by the Hosmer-Lemeshow test (*p* = 0.568). The following variables remained in the final model: women in a stable sexual relationship (OR = 14.21, 95%CI = 3.67–55.018), positive PCR for HPV (OR = 16.81, 95%CI = 4.19–67.42), *T. vaginalis* (OR = 8.566, 95%CI = 2.04–35.94) and *G. vaginalis* (OR = 6.13, 95%CI = 1.53–24.61) adjusted by age and qPCR for *U. parvum*.

**Fig. 1:**
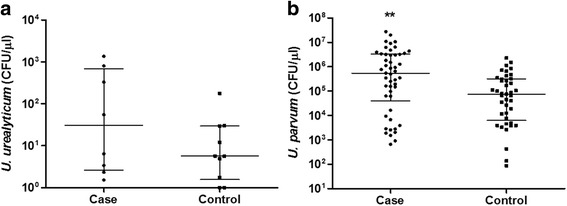
*U. urealyticum* and *U. parvum* DNA load quantification detected by qPCR (CFU/μL) from vaginal swab samples of women with and without cervical lesions. **a** Load of *U. urealyticum* (CFU/μL) among women with lesions (median = 30.6 CFU/μL) and without (median = 5.7 CFU/μL) (*p* = 0.2743) **b** Load of *U. parvum* (CFU/μL) among women with (median = 547,500 CFU/μL) and without lesions (median = 75,300 CFU/μL) (**p = 0.002). Median and interquartile range indicated by *solid lines* in the graph. Statistical analysis performed by Mann Whitney and was considered significant

To consider differences of the microorganism loads between the case and control groups, we considered only positive results, as shown in Fig. 1. *U. parvum* load showed a statistically significant difference in the Mann-Whitney U test (*p*-value = 0.002) between the case and control groups (Fig. 1).

## Discussion

In the Northeast region of Brazil, cervical cancer is the second most common cancer [2]. This region has the highest rates of poverty among Brazilian regions [11]. In this study, the demographic profile and sexual behavior of 132 women from Vitória da Conquista (a city in an arid region of the State of Bahia, Northeast Brazil) were evaluated. Besides this, HPV prevalence and other important sexual transmitted infectious agents were analyzed, as certain factors associated with the presence of cervical lesions.

In the present study, HPV prevalence was 53.8% (71/132) in the general population, 85.5% (53/62) in the case group and 25.7% (18/71) in the control group. In this study, 85.5% of cases with cervical lesions were positive for HPV. The viral DNA was detected in 24.5% of low-grade lesions (L-SIL/CIN I), 26.4% (CIN II) of intermediate-grade lesions and 49.1% of high-grade lesions (HSIL; CIN III/carcinoma in situ). In another case-control study conducted in Recife, Pernambuco, Brazil, women with squamous intraepithelial lesions, high-grade or cervical carcinoma comprised the case group, and women with normal cytology or benign cellular changes comprised the control group. Of the 610 women initially eligible, just 248 (40.7%) were PCR positive for HPV. Among these women, 29.0% (72/248) had a high-grade lesion [12]. Similarly, in Rio de Janeiro between 2000 and 2002, of 5833 samples obtained from cervical smear, 44.9% were positive for HPV, of which 14.1% of the HPV-positive had cervical lesions [13]. The Ludwig-McGill cohort study followed up women living in São Paulo (in Southeastern Brazil), between 1993 until 1997. The study was restricted to 1867 women who had completed all four visits within the first year, representing 76% of those enrolled in the study. Of these, 600 (32.1%) women tested positive for HPV infection on at least one of the four visits [14, 15].

Despite the high risk of developing lesions when infection with high-risk HPV occurs, 18 (25.7%) of 70 women with normal cytology were positive for HPV. This is the case because the samples can be positive for low-risk HPVs that are not responsible for the development of cervical lesions. Besides this, while infection with HPV is a necessary factor in the development of cervical cancer, not all positive cases of virus infection will result in cancer. Furthermore, this virus is commonly detected in the population. In routine cytological examination among women with normal cytology, about 10–30% are positive for any HPV type [3, 16]. In a review by Baseman and Koutsky, it was observed that this percentage ranges from 2 to 44%, depending on the regions analyzed and which HPV detection tests were used [17].

The low rates of cervical cancer precursor lesions, added to high HPV prevalence in the general population and high HPV detection in patients with normal cytology supports the hypothesis that, although a necessary precursor, the presence of HPV alone is not sufficient for developing cancer. First, the development of cervical lesions depends on infection by high risk Human Papillomavirus (HPV), such as HPV-16 and HPV-18. HPV-related carcinogenesis depends on different factors such as HPV type, virus persistence, sustained viral oncogene expression, viral load and viral genome integration. These HPV types express the oncoproteins E6 and E7, which interfere with cellular machinery inhibiting cell death, promoting cell immortalization, and after cell transformation [3, 18]. Furthermore, other factors have been linked with the development of cervical lesions, including co-infection with other Sexually Transmitted Agents (STA) [5]. The proposed mechanisms through which infectious agents might act as co-factors in HPV-associated tumorigenesis including direct biological interactions, such as modification of HPV replication and transcription, and indirect effects, such as inflammation and damage to the epithelial barrier that protects against HPV infection [19].

In this study, the detection of *Mollicutes*, *U. parvum* and its serotypes, *U. urealyticum*, *T. vaginalis, G. vaginalis*, *C. trachomatis* and *N. gonorrhoeae* was compared to determine a possible association with cervical lesions. There was a difference for both *U. parvum* and *U. urealyticum* between the prevalence observed in PCR and qPCR. These differences can be explained by the higher sensitivity of the qPCR technique compared with PCR, thus allowing a greater detection. Besides, the presence of *U. parvum* serotype 1 (OR = 0.111, 95% CI = 0.014–0.904) and serotype 6 (OR = 0.051, 95% CI = 0.007–0.397), in first instance, has been verified as a protective factor for the cervical lesions development. However, when comparing the number of positive samples for *U. parvum* (32 cases and 35 controls) and the total number of serotypes obtained by adding all positive samples from serotypes 1, 3/14 and 6 (13 cases and 36 controls) we observe that there is no match between the results. This fact, added to the disparity between the results detected by PCR and qPCR, demand for future studies with more sensitive techniques, like qPCR, to verify the real association of such serotypes with the development of cervical lesions.

In addition to the observed prevalence, a positive correlation between *U. parvum* and cervical lesions in the univariate analysis was detected. However, after logistic regression, the presence of *U. parvum* lost its association with cervix lesions. There are few and contrasting data in the literature on the relationship between cervical lesion and ureaplasma presence. Ekiel and collaborators conducted a similar study and evaluated 182 women with changes in the cervix: 67 women with atypical squamous cells of undetermined significance (ASCUS), 49 women with LSIL, 22 with HSIL, 39 with normal cytology and 5 with invasive cancer [8]. Sixty-four (35.2%) were positive for *Mollicutes*, but 58 (90.7%) were positive for *U. parvum* and nine (14.1%) for *U. urealyticum*. They also found that the ureaplasmas were more frequent among women with HSIL. In another study by Lukic et al., 239 women with different disorders in the cervical region were studied: 66 with ASCUS, 115 with low-grade intraepithelial lesions (LSIL) and 58 with high-grade intraepithelial lesions (HSIL). They did not diagnose *U. parvum*; however, they found that among women diagnosed with HSIL, 26 (45%) were positive for *U. urealyticum* [20]. In this study, there was a higher detection rate of *U. parvum* than *U. urealyticum* among women with HSIL. According to Kong et al., *U. parvum* is more common in clinical isolates of the genital region than *U. urealyticum* [21].

After analyzing the load of *U. parvum*, patients with cervical lesions showed higher *U. parvum* load (median = 547,500 colony-forming unit (CFU)/μl) than those without cervical lesions (median = 75,300 CFU/μl) (*p*-value ≤ 0.002). This can be explained because more microorganisms in the local area could intensify the inflammatory response and/or activate some virulence factor of the pathogen. This could favor infection progression and the development of cervical lesions. Few studies have been conducted focusing on the association of ureaplasma load with disorders of the reproductive system, especially with precursor cervical lesions of cervical cancer [22]. Lu et al. compared *U. parvum* and *U. urealyticum* load in women who had cervicitis, and found a positive relationship between the risk of microbial load and cervicitis [23]. Another study found an association between higher load *U. urealyticum* and the presence of non-gonococcal urethritis and non-chlamydial (NGU-NC) in men but did not observe the same for *U. parvum* [24]. The causal relationship between the load and ureaplasmas in this disease was also verified in other studies [25, 26]. However, there were no statistically significant differences for *U. urealyticum* load among women with and without cervical lesion.

Among the four serotypes of *U. parvum*, 3/14 showed greater detection of all serotypes, as described in the literature [8, 21], but did not show a statistically significant relation between women with or without lesions. However, *U. parvum* serotypes 1 and 6 were more frequent among women with lesions, and were inversely associated with the presence of cervical lesions. Furthermore, since the detection of the serotypes was obtained by PCR, it was not possible to observe the microbial load of each of the serotypes in the samples, since the presence of high/low load of microorganisms in a clinical specimen is associated with a stronger inflammatory response and some diseases [22, 23, 25–27]. Studies should be extended to define the differences between the genes capable of causing variations in the pathogenicity mechanisms of these ureaplasmas, which may help explain precisely why high bacteria loads were related with cervical lesions.

It is believed that the inflammatory response caused by the heavy load of microorganisms at the site of infection lead to cell damage suggestive of cervical lesions, as has been observed for other microorganisms. Other sexually transmitted infectious agents were detected. *T. vaginalis* was detected in 61.3% of women who had lesions. There was a greater risk related to presence of this agent and the development of cervical lesions. Zhang et al. also reported a positive association between *T. vaginalis* and the presence of cervical lesion. A total of 16,797 residents in the Jingan district, Shanghai, China, were studied and followed from 1974 to 1985. A risk of 3.31 was found (95% CI = 1.5 to 7.4) [28]. Moreover, Lukic and co-workers (2006) detected low levels of this microorganism among women tested. Only 1% of women with LSIL were positive for *T. vaginalis*, and among those with no HSIL detection, and there was no statistically significant association [20]. Verteramo co-workers (2009) also reported low detection of this microorganism (1.16%) [29]. In this study, *G. vaginalis* was detected in 91.9% of all women who had lesions; a positive association with the presence of injury was also found, showing a higher detection among those with HSIL [29]. In a study conducted in the Netherlands from 1991 to 2006, cervical samples from 800,498 women were obtained, and a positive association was found between the presence of the organism and the presence of cervical injury [30]. *C. trachomatis* was detected in only 3.2% of women and *N. gonorrhoeae* was not detected in women with cervical lesions. In this study, these two microorganisms were not associated with cervical lesions possibly due to the low number of cases included in this study. Thus, further studies are needed to better understand the association of these microorganisms with the presence of cervical cancer.

In this study, women living in the countryside and with an education level below elementary education, showed to be at greater risk for developing cervical lesions. In Recife, a city of the State of Pernambuco, Brazil [12], women who live in rural areas and have less than 4 years of elementary school also have a greater risk for developing cervical cancer. Herein young women with a low literacy level living in the countryside probably had less knowledge about the importance of cervical screening and less access to healthcare. The lack of association between race/color and the occurrence of HSIL/cancer certainly reflects the extensive racial miscegenation in the country, especially in the Northeast and in the population covered by the Public Health System (SUS – Brazil).

Women with irregular sexual activity (widows, divorced, or having occasional sexual intercourse) were less likely to develop cervical lesions compared to those in a stable relationship (married, dating or engaged, or maintaining a stable relationship). Women in stable relationship, in interview moment, have declared being with only one partner. Thus, one possible explanation to this unexpected fact is that many partners do not use condoms, and if they are involved in extramarital relationships, they are at great risk to contract HPV virus and infect their wives [31]. However, this factor is difficult to evaluate because our data collection represents one data point, not data over time. Thus, there was no monitoring of patients to understand if the women used condoms or not in all relationships, since only one contact with a carrier virus partner is enough to be infected, and this infection may or may not progress to lesions years later.

## Conclusion

Even if the role of HPV is evident in the development of cervical cancer, further studies are needed to better understand the complexity and multifactorial profile of this condition. In this study, variables such stable relationship, HPV, *T. vaginalis, G. vaginalis* were associated with cervical lesions in epidemiological studies. *U. parvum* load was higher in woman with cervical lesions compared with women without lesion. Despite the evidence presented in this paper on the role of ureaplasmas, *T. vaginalis* and *G. vaginalis* in cervical lesions, longitudinal studies with a larger population must be done to precisely define their association with the emergence of cervical injury. In addition, conducting infection tests in in vitro and in vivo study models will further elucidate virulence factors involved in the establishment of cervical lesions. These data are unprecedented for the population under study, and may aid local health authorities in improving prevention measures, and treatment, in order to improve the quality of life of women in the municipality of Vitória da Conquista, Bahia, Brazil. When all aspects involved in pathogenesis are better understood, this knowledge may contribute to improving prevention strategies and treatment.

## Methods

### Study design and participants

The study described here is a transversal study of cervical lesions in women (case-control), carried out from 2011 to 2013. One hundred and forty women were initially included (70 individuals with cervical lesions and 70 without lesions). The sample size was determined with statistical power of 95% and 0.05 statistical significance using the reports of cervical lesions and total size of women from Vitória da Conquista (158,987 women/306,866 total inhabitants – IBGE 2010). The cases (women with cervical lesion) were from the Family Health Center CAE II of Public Health System (SUS – Brazil) located in Vitória da Conquista city, Bahia, Brazil. Women seeking medical consultations, with a positive diagnosis for cervical lesions (Cervical Intraepithelial Neoplasia (CIN) 1, 2 and 3 or in situ carcinoma) were selected as the case group. The controls (women without cervical lesion) were from the Primary Care Units of Public Health Clinics (SUS – Brazil) also located in Vitória da Conquista city, Bahia, Brazil. Women with a negative diagnosis were selected for the control group. Screening was conducted among patients aged from 14 to 78 years. Then 140 women (70 cases and 70 controls) initially answered a questionnaire to provide a socioeconomic and sexual activity profile. This study was previously approved by the Ethics Committee on Human Research of the Institute of Biomedical Science – University of São Paulo, Brazil, (protocol number 1089).

### Detection of sexually transmitted agents

Cervical samples were taken by cytobrush and swab from the ectocervix and from the endocervix for detecting HPV DNA and bacteriological tests. Swabs were transferred to a tube containing a transport medium [32].

#### HPV detection

For HPV detection, two microtubes of each sample were sent to the HPV laboratory of the Santa Casa Research Institute of São Paulo (São Paulo, Brazil). We extracted DNA from all cervical specimens using digestion with 100 μg/mL of proteinase K for 3 h at 55 °C, followed by organic extraction and ethanol precipitation. Extracted DNA in aqueous solution was quantified (ratios 260/280 and 260/230) by NanoDrop 2000 Spectrophotometer (Thermo-Fisher Scientific®, Waltham/MA/USA). DNA concentrations were normalized to 50 ng/μl to perform β-globin PCR (primers and references are described in Additional file 1: Table S1).

For β-globin PCR, we used 1X buffer (Buffer – 10× Tris-HCl (pH 8.4), 500 mM KCl), 0.2 mM of deoxiribonucleotide triphosphate (dNTP), 2.0 mM MgCl_2_, 80 nM of each primer G73/G74 (described in Additional file 1: Table 1), 0.5 U of Taq DNA polymerase (Invitrogen®, São Paulo, SP, Brazil), 3.0 μL of the DNA (50 ng/μL) in a final volume of 25 μL. The assays were performed in a Veriti Thermal Cycler (Applied Biosystems, São Paulo, SP, Brazil). The PCR was conducted with an initial denaturation of 95 °C for 5 min, followed by 40 cycles of 95 °C for 1 min, annealing temperature of 55 °C for 1 min, extension temperature of 72 °C for 1 min, a final cycle of 72 °C for 5 min and a hold step at 4 °C. This protocol amplifies a DNA segment of 268-bp (Additional file 1: Table S1). Eight patients were excluded from the case group because they were negative for the β-globin. Other samples presented positive results for β-globin, ensuring DNA quality for the following testing. Then, 132 samples (62 cases and 70 controls) were tested for the presence of HPV DNA.

For the HPV PCR, we used 1X buffer (Buffer – 10× Tris-HCl pH 8.4, 500 mM KCl), 0.2 mM of deoxiribonucleotide triphosphate (dNTP), 4.0 mM MgCl_2_, 1 μM of each primer GP5/GP6 (described in Additional file 1: Table S1), 0.5 U of Taq DNA polymerase (Invitrogen®, São Paulo, SP, Brazil), 3.0 μL of the DNA (50 ng/μL) in a final volume of 25 μL. The assays were performed in a Veriti Thermal Cycler (Applied Biosystems, São Paulo, SP, Brazil). The PCR was conducted with an initial denaturation of 94 °C for 5 min, followed by 39 cycles of 95 °C for 1 min, annealing temperature of 40 °C for 2 min, extension temperature of 72 °C for 1.5 min, a final cycle of 72 °C for 7 min and a hold step at 4 °C. This protocol amplifies a highly conserved 154-bp segment in the L1 viral gene (flanked by primers GP5/6) (Additional file 1: Table S1).

As positive control for all PCR, HeLa (an HPV 18 positive cervical carcinoma cell line; ATCC - CCL-2) cells were used. The products of the targeted amplified DNA and a standard molecular weight of 100 bp were electrophoresed on 1.0% agarose gel stained with 5 μL of GelRed 10,000X (Biotium, Fremont Ca, USA). Then the gels were photodocumented.

#### Pathogen detection

For bacteriological tests, genomic DNA samples of vaginal swabs were obtained according to the recommendations of Purelink™ Genomic DNA Mini Kit (Invitrogen, São Paulo, SP, Brazil). DNA was extracted from two microtubes of each sample for detecting *Ureaplasma urealyticum* (UU) and *Ureaplasma parvum* (UP), *Trichomonas vaginalis* (TV), *Gardnerella vaginalis* (GV), *Chlamydia trachomatis* (CT) and *Neisseria gonorrhoeae* (NG).

The DNA solutions from clinical samples were subjected to conventional PCR and qPCR. The PCR was performed in a microtube (0.2 mL) adding: 1X buffer (Buffer - 200 mM Tris-HCl pH 8.4, 500 mM KCl), 0.2 mM of deoxiribonucleotide triphosphate (dNTP), 2.0 mM MgCl_2_, 50 pmol of each primer (described in Additional file 1: Table S1), 1.0 U of Taq DNA polymerase (Invitrogen®, São Paulo, SP, Brazil), 1.0 μL of the DNA from a PureLink extraction kit in a final volume of 50 μL. Positive (DNA of microbial strains) and negative (without DNA) controls were included. The assays were performed in a Veriti Thermal Cycler (Applied Biosystems, São Paulo, SP, Brazil). The PCR was conducted with an initial denaturation of 94 °C for 10 min, followed by 35–40 cycles of 94 °C for 30 s, the annealing temperature was set according to each primer (Additional file 1: Table S1), extension temperature of 72 °C for 45 s, a final cycle of 72 °C for 10 min and a hold step at 4 °C.

As positive controls, we used strains of *Ureaplasma urealyticum* (UU) and *Ureaplasma parvum* (UP) stored in our laboratory. These strains were growth in UB medium [33]. Samples of *Trichomonas vaginalis* (TV), *Gardnerella vaginalis* (GV), *Chlamydia trachomatis* (CT) and *Neisseria gonorrhoeae* (NG) were gently seeded by Dr. Jorge Sampaio, (Fleury Institute/São Paulo – Brazil) and directly submitted to DNA extraction. The products of the targeted amplified DNA and a standard molecular weight of 100 bp were electrophoresed on 1.0% agarose gel with ethidium bromide (10 mg/ml). Then the gels were photodocumented.

We used quantitative PCR (qPCR) to measure the *U. urealyticum* and *U. parvum* DNA load in clinical samples. The assays were performed in duplicate with a Real-Time PCR System 7300 Applied Biosystems platform and TaqMan probes, following the manufacturer’s basic amplification protocol. For constructing DNA standards for absolute quantitation, the ureaplasmas were first cultured in 2 ml at 37 °C and expanded to a 50 ml of UB broth. In a logarithmic growth phase (based in colorimetric changes), the culture was centrifuged at 20,600 g for 30 min at 25 °C. The DNA was extracted using a PureLink™ Genomic DNA Mini Kit. The genomic DNA copy number was calculated relating the amount of DNA (ng) measured by spectrophotometry (NanoDrop ND-1000, Witec Ag, Littau, Switzerland) with the length of DNA (bp) of the respective strains. Then, 10-fold serial dilutions (10^7−^1 copies/μl) of the ureaplasma DNA standard were prepared and analyzed. The reagents used were: 12.5 μL of Master Mix (Applied Biosystems, São Paulo, SP, Brazil), 0.75 μL of each primers diluted to 10 pmol (described in Additional file 2: Table S2), 0.5 μL probe 5 mM, 1.0 μl of DNA and 9.5 μL of water to a final volume of 25 μL. The samples were submitted to amplification in StepOne software (Applied Biosystems) under the following cycles: 50 °C for 2 min, 95 °C for 10 min, followed by 40 cycles of 95 °C for 15 s and 58 °C for 30 s (primers and probes, Additional file 2: Table S2). For each reaction, we added a new curve to the system, with r2 ≥ 0.950 being set for a good analysis; efficiency from 95 to 105% and slope close to −3.2 to −3.6. Data was analyzed and the threshold of each sample was determined with the aid of StepOne™ v2.1 software (Applied Biosystems, São Paulo, SP, Brazil). The Ct number of each sample was compared.

### Statistical analyses

Categorical variables were shown as frequencies and percentages. In order to evaluate factors associated with cervical lesions, we used the logistic regression model and an odds ratio (OR) and respective 95% confidence intervals (95%CI) were calculated. The selection of variables was done by a stepwise method and OR unadjusted and adjusted were shown in the final model. The goodness of the fit was verified by Hosmer-Lemeshow’s test. Finally, only variables with a *p*-value less than 0.05 were used in the final model, unless the variable was biologically important. The microbial load of case and control groups was compared using the nonparametric Mann-Whitney. Significance level was 5% in all hypotheses tested. All analyses were evaluated by SPSS 20.0 software (SPSS Inc., Chicago, USA), R software (R Core Team, 2015) and the GraphPad Prism version 5.00 (GraphPad Software, San Diego, California, USA).

## Additional files

Additional file 1: Table S1. Primers used in PCR. (DOC 89 kb)

Additional file 2: Table S2. Primers and probes used in qPCR to quantify DNA of *U. urealyticum* and *U. parvum* in cervical samples. (DOC 50 kb)

## Abbreviations

ASCUS: Atypical Squamous Cells of Undetermined Significance
BV: Bacterial Vaginosis
CFU: Colony-Forming Unit
CI: Confidence Intervals
CIN: Cervical Intraepithelial Neoplasia
E6: E6 oncoprotein
E7: E7 oncoprotein
HPV: Human Papillomavirus
HSIL: High-grade Intraepithelial Lesions
IL-1: Interleukin 1
IL-6: Interleukin 6
LSIL: Low-grade intraepithelial lesions
NGU-NC: Non-Gonococcal and Non-Chlamydial Urethritis
OR: Odds ratio
PCR: Polymerase Chain Reaction
qPCR: Quantitative Polymerase Chain Reaction
SD: Standard deviation
STA: Sexually Transmitted Agents
SUS: Public Health System of Brazil
TNF-α: Tumour Necrosis Factor alpha
