# Supplementary material for: Co-infection of sexually transmitted pathogens and Human Papillomavirus in cervical samples of women of Brazil
Source: BMC Infect Dis. 2017 Dec 15;17:769. doi: 10.1186/s12879-017-2835-5 (PMC5732421; doi:10.1186/s12879-017-2835-5)
Supplement: Supplementary file 1 — Primers used in PCR. (DOC 89 kb) [file 12879_2017_2835_MOESM1_ESM.doc]

**Supplementary table 1:** Primers used in PCR.

| Description | Primers | Sequences | Amplicons | References |
| --- | --- | --- | --- | --- |
| *Mollicutes* | GPO3 | 5' GGGAGCAAACACGATAGATACCCT 3' | 270 pb |  |
|  | MGSO | 5' TGCACCATCTGTCACTCTGTTAACCTC 3’ |  |  |
| *U. urealyticum* | UMS170 | 5' GTATTTGCAATCTTTATATGTTTTCG 3' | 476 pb |  |
|  | UMA263 | 5' TTTGTTGTTGCGTTTTCTG 3' |  |  |
| *U. parvum* | UMS57 | 5' TAAATCTTAGTGTTCATATTTTTTAC 3' | 327 pb |  |
|  | UMA222 | 5' GTAAGTGCAGCATTAAATTCAATG 3' |  |  |
| *U. parvum*, serotype 1 | UMS 83 | 5′ TACTGATAGAAATTATGTAAGATTGC 3′ | 398 pb |  |
|  | UMA 269A | 5′ CCAAATGACCTTTTGTAACTAGAT 3′ |  |  |
| *U. parvum,* serotypes 3/14 | UMS 125 | 5′ GTATTTGCAATCTTTATATGTTTTCG 3′ | 442 pb |  |
|  | UMA 269B | 5′ CTAAATGACCTTTTTCAAGTGTAC 3′ |  |  |
| *U. parvum,* serotype 6 | UMS 54 | 5′ CTTAGTGTTCATATTTTTTACTAG 3′ | 369 pb |  |
|  | UMA 269A | 5′ CCAAATGACCTTTTGTAACTAGAT 3′ |  |  |
| *Neisseria gonorrhoeae* | HO1 | 5' GCTACGCATACCCGCGTTGC 3' | 390 pb |  |
|  | HO3 | 5' CGAAGACG1TCGAGCAGACA 3' |  |  |
| *Gardnerella vaginalis* | GV1 | 5’ TTACTGGTGTATCACTGTAAGG 3’ | 332 pb |  |
|  | GV3 | 5’ CCGTCACAGGCTGAACAGT 3’ |  |  |
| *Trichomonas vaginalis* | TVA5 | 5' GATCATGTTCTATCTTTTCA 3' | 102 pb |  |
|  | TVA6 | 5' GATCACCACCTTAGTTTACA 3' |  |  |
| *Chlamydia trachomatis* | KL1 | 5' TCCGGAGCGAGTTACGAAGA 3' | 241 pb |  |
|  | KL2 | 5' ATTCAATGCCCGGGATTGGT 3' |  |  |
| β-globin | G73 | 5' GAAGAGCCAAGGACAGGTAC 3' | 268 pb |  |
|  | G74 | 5' CAACTTCATCCACGTTCACC 3' |  |  |
| HPV | GP5 | 5' TTTGTTACTGTGGTAGATAC 3' | 154 pb |  |
|  | GP6 | 5' GAAAAATAAACTGTAAATCAT 3' |  |  |

**References:**

34. van Kuppeveld FJ, van der Logt JT, Angulo AF, van Zoest MJ, Quint WG, Niesters HG, Galama JM, Melchers WJ: **Genus- and species-specific identification of mycoplasmas by 16S rRNA amplification**. *Appl Environ Microbiol* 1992, **58**(8):2606-2615.

35. De Francesco MA, Negrini R, Pinsi G, Peroni L, Manca N: **Detection of Ureaplasma biovars and polymerase chain reaction-based subtyping of Ureaplasma parvum in women with or without symptoms of genital infections**. *European journal of clinical microbiology & infectious diseases : official publication of the European Society of Clinical Microbiology* 2009, **28**(6):641-646.

36. Ho BS, Feng WG, Wong BK, Egglestone SI: **Polymerase chain reaction for the detection of Neisseria gonorrhoeae in clinical samples**. *J Clin Pathol* 1992, **45**(5):439-442.

37. Zariffard MR, Saifuddin M, Sha BE, Spear GT: **Detection of bacterial vaginosis-related organisms by real-time PCR for Lactobacilli, Gardnerella vaginalis and Mycoplasma hominis**. *FEMS immunology and medical microbiology* 2002, **34**(4):277-281.

38. Riley DE, Roberts MC, Takayama T, Krieger JN: **Development of a polymerase chain reaction-based diagnosis of Trichomonas vaginalis**. *Journal of clinical microbiology* 1992, **30**(2):465-472.

39. Mahony JB, Luinstra KE, Jang D, Sellors J, Chernesky MA: **Chlamydia trachomatis confirmatory testing of PCR-positive genitourinary specimens using a second set of plasmid primers**. *Molecular and cellular probes* 1992, **6**(5):381-388.

40. Campos EA, Simões JA, Rabelo-Santos SH, Sarian LO, Pitta DR, Levi JE, Derchain S: **Recovery of DNA for the detection and genotyping of human papillomavirus from clinical cervical specimens stored for up to 2 years in a universal collection medium with denaturing reagent**. *Journal of virological methods* 2008, **147**(2):333-337.

41. Gravitt PE, Peyton CL, Alessi TQ, Wheeler CM, Coutlée F, Hildesheim A, Schiffman MH, Scott DR, Apple RJ: **Improved amplification of genital human papillomaviruses**. *Journal of clinical microbiology* 2000, **38**(1):357-361.

42. Cao X, Wang Y, Hu X, Qing H, Wang H: **Real-time TaqMan polymerase chain reaction assays for quantitative detection and differentiation of Ureaplasma urealyticum and Ureaplasma parvum**. *Diagnostic microbiology and infectious disease* 2007, **57**(4):373-378.
