# Supplementary material for: Co-infection of sexually transmitted pathogens and Human Papillomavirus in cervical samples of women of Brazil
Source: BMC Infect Dis. 2017 Dec 15;17:769. doi: 10.1186/s12879-017-2835-5 (PMC5732421; doi:10.1186/s12879-017-2835-5)
Supplement: Supplementary file 2 — Primers and probes used in qPCR to quantify DNA of U. urealyticum and U. parvum in cervical samples. (DOC 50 kb) [file 12879_2017_2835_MOESM2_ESM.doc]

**Supplementary table 2:** Primers and probes used in qPCR to quantify DNA of *U. urealyticum* and *U. parvum* in cervical samples

| **Description** | **Primers** | **Sequences** | **Reference** |
| --- | --- | --- | --- |
| *U. urealyticum* | UUureF | 5' ATCGACGTTGCCCAAGGGGA 3' |  |
|  | UUureR | 5' TTAGCACCAACATAAGGAGCTAAATC 3' |  |
|  | Probe | 5'-FAM-TTGTCCGCCTTTACGAG-MGB-3' |  |
| *U. parvum* | UPureF | 5'CATTGATGTTGCACAAGGAGAAA 3' |  |
|  | UPureR | 5' TTAGCACCAACATAAGGAGCTAAATC 3' |  |
|  | Probe | 5'-FAM-TTGACCACCCTTACGAG-MGB-3' |  |
